# Polylysine as a functional biopolymer to couple gold nanorods to tumor-tropic cells

**DOI:** 10.1186/s12951-018-0377-7

**Published:** 2018-05-31

**Authors:** Claudia Borri, Sonia Centi, Fulvio Ratto, Roberto Pini

**Affiliations:** 1 Institute of Applied Physics ‘N. Carrara’, National Research Council of Italy, Via Madonna del Piano, 10, 50019 Sesto Fiorentino, Italy; 2 Department of Experimental and Clinical Biomedical Sciences ‘M. Serio’, University of Florence, Largo Brambilla, 3, 50134 Florence, Italy

**Keywords:** Gold nanorods, Macrophages, Cellular vehicles, Cell migration, Cytokine release

## Abstract

**Background:**

The delivery of plasmonic particles, such as gold nanorods, to the tumor microenvironment has attracted much interest in biomedical optics for topical applications as the photoacoustic imaging and photothermal ablation of cancer. However, the systemic injection of free particles still crashes into a complexity of biological barriers, such as the reticuloendothelial system, that prevent their efficient biodistribution. In this context, the notion to exploit the inherent features of tumor-tropic cells for the creation of a Trojan horse is emerging as a plausible alternative.

**Results:**

We report on a convenient approach to load cationic gold nanorods into murine macrophages that exhibit chemotactic sensitivity to track gradients of inflammatory stimuli. In particular, we compare a new model of poly-l-lysine-coated particles against two alternatives of cationic moieties that we have presented elsewhere, i.e. a small quaternary ammonium compound and an arginine-rich cell-penetrating peptide. Murine macrophages that are exposed to poly-l-lysine-coated gold nanorods at a dosage of 400 µM Au for 24 h undertake efficient uptake, i.e. around 3 pg Au per cell, retain the majority of their cargo until 24 h post-treatment and maintain around 90% of their pristine viability, chemotactic and pro-inflammatory functions.

**Conclusions:**

With respect to previous models of cationic coatings, poly-l-lysine is a competitive solution for the preparation of biological vehicles of gold nanorods, especially for applications that may require longer life span of the Trojan horse, say in the order of 24 h. This biopolymer combines the cost-effectiveness of small molecules and biocompatibility and efficiency of natural peptides and thus holds potential for translational developments.

**Electronic supplementary material:**

The online version of this article (10.1186/s12951-018-0377-7) contains supplementary material, which is available to authorized users.

## Background

Over recent years, the notion to use tumor-tropic cells as a biological taxi to take up and deliver functional particles to solid tumors has attracted interest as a powerful solution to overcome all biological barriers that exist along the way from the bloodstream to the tumor microenvironment (TME) [1, 2]. In order to implement this solution, relevant steps include the identification and harvesting of a population of tumor-tropic cells, their coupling to a formulation of passenger particles in vitro, and their injection into an oncological patient, with the mission to carry their cargo to her/his tumor site(s) by virtue of their chemotactic functions. This approach represents a radical alternative to a systemic administration of free particles, which remains an outstanding issue [3], where capture by the reticuloendothelial system regularly prevents most of the injection dose to reach the TME, in spite of the EPR (enhanced permeability and retention) effect [4–7] or even upon active targeting [8–11]. Examples of tumor-tropic cells that may serve as a biological taxi include immune-system cells and stem cells that undergo recruitment during cancer progression [12–16]. Another key advantage of these cells is their native ability to infiltrate the hypoxic core of a tumor site, which is hardly accessible by free particles, due to its inherent lack of vasculature [17, 18]. On the other hand, the implementation of a cell-based delivery system implies an intimate contact of the passenger particles with their biological taxi, which should remain functional for hours or days, rather than the malignant cells. That is why this approach is most appropriate to deliver inert particles that convey contrast for applications as the photoacoustic imaging [19–22] and optical hyperthermia of cancer [23–26], which hold spatial resolution coarser than the single cell and are exempt of the final steps of the so-called CAPIR cascade (Circulation, Accumulation, Penetration, Internalization, and drug Release [3]). In this context, several authors have proposed different models of biological vehicles of plasmonic particles, including macrophages [27–29], T lymphocytes [30, 31], mesenchymal [32, 33] and neural [34, 35] stem cells or endothelial progenitors [36].

Gold nano-crystals are nowadays available in a remarkable variety of sizes and shapes, which enables a rich selection of their colloidal and plasmonic features [20, 37, 38]. In particular, gold nanorods represent a convenient choice, owing to their ease of synthesis and modulation of their longitudinal band of plasmonic oscillations in the near-infrared window of principal interest in biomedical optics [39–43].

Here, we address the modification of gold nanorods for their efficient and harmless coupling to tumor-tropic cells, which should retain their tumor-homing and pro-inflammatory profiles, in order to serve as a biological taxi. A robust strategy to promote the cellular uptake of these particles combines their termination with biopolymers that provide for steric stabilization, such as polyethylene glycol (PEG) [44, 45], and cationic moieties [29, 34, 35, 46] that are prone to interact with the anionic phospholipids on plasmatic membranes [47–49] and/or to induce receptor-mediated endocytic and phagocytic pathways [50, 51]. In our recent work, we have disclosed a modification of gold nanorods with PEG and mercaptoundecyl trimethyl ammonium bromide (MUTAB) that provides high efficiency and reproducibility of phagocytosis from tumor-tropic macrophages [28, 29]. However, quaternary ammonium compounds, such as MUTAB, are likely to cause cytotoxicity and so require caution [52–55]. More recently, we have reported on an alternative design that replaces these compounds with biocompatible arginine-rich cell penetrating peptides (CPPs) [56]. How natural CPPs really work remains a matter of debate but seems to relate to their abundance of cationic or lipophilic residues [57–60]. This work starts from the consideration that, if the worth of synthetic arginine-reach CPPs was really limited to their cationic backbone, their cost–benefit profile would be rather poor. In an attempt to identify a translational solution that may combine the cost-effectiveness of MUTAB and biocompatibility of CPPs, we took inspiration from the pool of biopolymers in use in cell biology. In particular, poly-l-lysine (pLys) is a common solution to coat plastic- and glassware to stimulate the adhesion of most cells. In addition, pLys has already been used as a cross-linker to assemble various particles for imaging [61] or delivery of genetic material [62]. However, its use to couple functional particles to tumor-tropic cells has never been described, thus far.

In order to assess the feasibility of pLys-coated gold nanorods for integration into a biological taxi retaining tumor-homing and pro-inflammatory functions, we have benchmarked their parameters against those of their MUTAB- and CPP-coated predecessors. Our multi-parametric survey includes key features for a cell-based delivery system, such as the efficiency of uptake and retention of the passenger particles and the viability, migration and release of cytokines from the biological taxi, in the presence of relevant stimuli.

## Methods

### Materials

HAuCl_4_ (hydrogen tetrachloroaurate (III) hydrate), CTAB (hexadecyltrimethylammonium bromide), NaBH_4_ (sodium borohydride), ascorbic acid, silver nitrate, NHS (*N*-hydroxysuccinimide), EDC (1-ethyl-3-(3-dimethylaminopropyl)carbodiimide), MES (2-(*N*-morpholino)ethanesulfonic acid), acetic acid, sodium acetate, sodium chloride, DMSO (dimethyl sulfoxide), Tween^®^ 20, MUTAB, pLys and all other chemicals required for the preparation of all buffer solutions were purchased from Sigma-Aldrich Corporation (St. Louis, MO, USA), unless otherwise specified. Instead, m-PEG-SH [alpha-methoxy-omega-mercapto-poly(ethyleneglycol)] and c-PEG-SH (alpha-carboxy-omega-mercaptopoly(ethylene glycol)), M_w_ ≈ 5000 gmol^−1^ were acquired from Iris Biotech GmbH (Marktredwitz, Germany). CPPs were provided by Giotto Biotech Srl (Florence, Italy) [56]. All components for cell cultivation were obtained from Euroclone SpA (Pero, Italy). Instead, LPS (bacterial lipopolysaccharide), recombinant mouse chemokine MIP-1α (CCL-3), BSA (bovine serum albumin), PFA (paraformaldehyde) and Harris’ hematoxylin and eosin histological staining kit were also acquired from Sigma-Aldrich Corporation.

### Preparation of the particles

CTAB-capped gold nanorods were synthesized according to the autocatalytic reduction of HAuCl_4_ with ascorbic acid that was proposed by Nikoobakht et al. [63] and modified by Ratto et al. [40], with the intent to consume the full aliquot of HAuCl_4_ and gain more control over the total amount of gold in suspension. These particles were imaged with a CM12 transmission electron microscope (TEM) from Philips (Amsterdam, the Netherlands), with a voltage of 100 kV and operating in standard conditions. MUTAB- and CPP-coated gold nanorods were prepared according to the prescriptions of refs [29] and [56], respectively. Instead, pLys-coated gold nanorods were realized by the same protocol that was implemented for the preparation of their CPP-coated counterpart, with the replacement of CPPs with the same dose of pLys. Briefly, for MUTAB-coated particles, as-grown gold nanorods were transferred at a concentration of 1.6 mM Au into a 100 mM acetate buffer at pH 5 containing 500 µM CTAB and 50 ppm Tween^®^ 20, supplemented with 50 µM m-PEG-SH, left to react for 30 min at 37 °C, added with 500 µM MUTAB and left at rest for another 24 h at 37 °C. For CPP and pLys-coated particles, as-grown gold nanorods were transferred at a concentration of 1.6 mM Au into a saline (120 mM NaCl) 10 mM MES buffer at pH 6.5 containing 500 µM CTAB and 50 ppm Tween^®^ 20 and PEGylated by the addition of 45 µM m-PEG-SH and 5 µM c-PEG-SH for 2 h at 37 °C. Then, PEGylated gold nanorods were purified by 5 cycles of centrifugation and decantation, diluted to 800 µM Au in MES-saline, activated by the addition of 6 mM NHS and 24 mM EDC for 15 min at 37 °C and re-diluted to 400 µM Au in MES-saline containing 10 ppm CPPs or pLys, in order to achieve their coupling by amidation. Finally, all particles were purified by centrifugation and stored at a concentration of 4 mM Au in sterile phosphate buffered saline (PBS) at pH 7.4 at 4 °C until use. Their hydrodynamic size and electrokinetic potential were inspected by the use a Zetasizer nano ZS 90 platform from Malvern Instruments (Malvern, UK) and their optical extinction was analyzed by a V-560 spectrophotometer from Jasco (Tokyo, Japan).

### Cell line and culture conditions

J774a.1 murine macrophages were purchased from the American Type Culture Collection (ATCC^®^ TIB-67™, Manassas, VA, USA) and seeded on plastic culture flasks in Dulbecco’s Modified Eagle Medium (DMEM) supplemented with 10% fetal bovine serum, 1% l-glutamine and 1% penicillin–streptomycin solution. Cells were kept and left to grow under standard culture conditions (37 °C, 5% CO_2_).

### Measurement of cellular uptake and retention of the particles

The accumulation of gold nanorods in macrophages was quantified by an optical analysis. 5 × 10^5^ J774a.1 cells were plated in Petri dishes and exposed to 100 and 400 µM Au pLys-coated gold nanorods from 1 to 48 h, MUTAB-coated gold nanorods from 30 min to 24 h and CPP-coated gold nanorods from 2 to 32 h in serum-free medium (SFM). At the end of these treatments, particles were accurately removed and macrophages were sequentially fixed with a solution of 3.6% PFA in PBS for 10 min at room temperature, washed with PBS, harvested, centrifuged for 5 min at 1000 rpm, re-suspended in 120 μl PBS in a quartz micro-cuvette and directed to an optical inspection with a V-560 spectrophotometer from Jasco.

In order to assess the effect of exocytic release on the retention of the passenger particles subsequent to their internalization, macrophages were treated with 100 and 400 µM Au gold nanorods for 24 h and then cultured in fresh SFM from 24 to 48 h, fixed and prepared for the optical analysis as is mentioned above.

In order to quantify the amount of particles taken up per cell, the spectra of optical extinction were modeled as a linear combination of separate contributions from the macrophages and the gold nanorods. In particular, the former was recovered from an empirical measurement of a standard population of macrophages. The latter was devised as a numerical approximation of the plasmonic band from an ensemble of gold nanorods, which was described as an integral over Gans lineshapes [39], by using the dielectric function of gold by Etchegoin et al. [64] and was also subject to an empirical calibration. With these calibrations, the analysis of the experimental spectra returns a number density of cells in cm^−3^ and a density of gold nanorods in ppm or µg cm^−3^, which are then combined to achieve a mass of gold per cell. Details and a demonstration of this method are provided elsewhere [65, 66].

### Measurement of cytotoxicity of the particles

Cell viability was assessed by a WST-8 assay (Cell counting kit-8, Sigma-Aldrich Corporation, St. Louis, MO, USA). The highly water-soluble tetrazolium salt WST-8 is reduced into formazan by dehydrogenases in cells and the optical absorbance of formazan at the wavelength of 450 nm is proportional to the number of living cells.

8 × 10^3^ macrophages were cultured in 96-well plates and incubated with 50–400 μM Au pLys-coated gold nanorods from 16 to 72 h and with MUTAB- and CPP-coated gold nanorods from 4 to 96 h. Each sample was prepared in triplicate. At the end of these treatments, each well was washed with PBS and added with 100 µl SFM supplemented with 10% WST-8 reagent for 2–4 h at 37 °C. The concentration of formazan was quantified by a LT-4000 microplate reader from Labtech (Bergamo, Italy) at the wavelength of 450 nm with a reference wavelength of 630 nm and subtracting blank values. Data were expressed as percent of optical absorbance with respect to controls.

In order to assess the viability of the biological taxi subsequent to its loading, cells were treated with 50–400 μM Au pLys-coated gold nanorods and with 100–400 μM Au MUTAB- and CPP-coated gold nanorods for 24 h. Each sample was prepared in triplicate. Then particles were accurately removed and macrophages were carefully washed with PBS and incubated with fresh SFM. After another 24–48 h from removal of the particles, the WST-8 assay was performed as above.

### Measurement of chemotaxis of the macrophages

Cell migration was investigated by the use of 24-well plates with 8 µm-pore polycarbonate membrane inserts (Transwell^®^ Permeable Supports, Costar^®^, Corning Life Sciences, Lowell, MA, USA). Confluent macrophages were cultured in SFM and then treated with 100 and 400 μM Au gold nanorods for 24 h. After harvesting, 2.5 × 10^4^ cells were re-suspended in 100 μl SFM and added to the upper side of the Transwell^®^ inserts. The lower side of the wells was filled with 600 μl SFM with or w/out 50 ng/ml MIP-1α chemokine, in the presence of .1% BSA. Then plates were incubated overnight at 37 °C. After this incubation, non-migrated cells on the upper surface of the inserts were removed with a cotton swab. Migrated cells were fixed with a solution of 3.6% PFA in PBS for 10 min at room temperature and stained with Harris’ hematoxylin and eosin. Inserts were observed by an optical microscope at 40× magnification and migrated cells were quantified as mean ± SD in five random fields.

### Measurement of production of pro-inflammatory cytokines

The release of interleukin-6 (IL-6), tumor necrosis factor-α (TNF-α) and interleukin-1β (IL-1β) in supernatants of the macrophages was evaluated by ELISA (Thermo Scientific, Pierce Biotechnology, Rockford, IL, USA), according to the instructions of the manufacturer of the kits. Briefly, J774a.1 cells were treated with 100 and 400 μM Au gold nanorods for 24 h. Then particles were accurately removed and macrophages were carefully washed with PBS and activated with LPS for 24 h. Aliquots of the supernatants of the cell cultures were collected and assayed for a quantitative measurement of the production of murine cytokines.

## Results and discussion

### Cellular uptake and retention of the particles

**Fig. 1:**
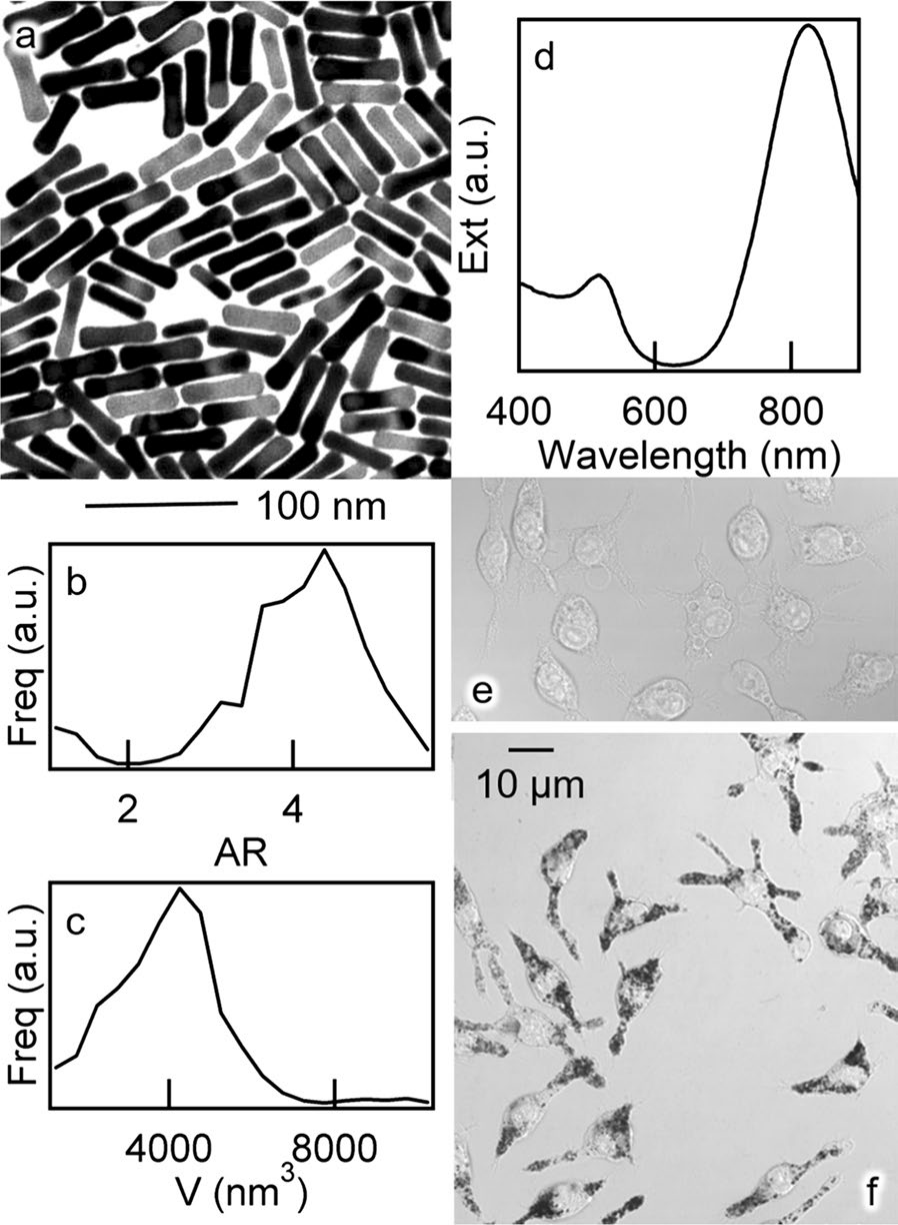
**a** Representative (300 × 300) nm^2^ TEM micrograph of as-synthesized gold nanorods. **b**, **c** Relevant volumetric distributions of aspect ratios and volumes derived from the analysis of around 500 particles. **d** spectrum of optical extinction of a suspension of pLys-coated gold nanorods. **e**, **f** Representative images of untreated controls and macrophages exposed to 400 µM Au pLys-coated gold nanorods for 24 h, from a confocal microscope (TCS SP8, Leica Microsystems, Heidelberg, Germany) operated in transmission mode at 63× magnification

The left-hand panels of Fig. 1 show a representative TEM micrograph of as-synthesized particles and their analysis in terms of volumetric distributions of aspect ratios and volumes. Gold nanorods display a typical diameter of (13 ± 2) nm and length of (55 ± 8) nm, which convey an aspect ratio of 4.2 ± .9 and volume of (4000 ± 1700) nm^3^. Figure 1d shows a vis–NIR spectrum of optical extinction of pLys-coated particles. The plasmonic feature around 800 nm is a distinctive hallmark of gold nanorods with an aspect ratio around 4 [39, 42] and underwent no variation upon PEGylation and bio-conjugation, as was reported for MUTAB- [29], CPP- [56] as well as sulfonamide- [66] and IgG- [67] coated gold nanorods that were protected with PEG. With respect to as-synthesized gold nanorods, the hydrodynamic size of pLys-coated particles increased by around 20 nm, i.e. from around 50 to 70 nm. Instead their electrokinetic potential in ultrapure water decreased by almost 40 mV, i.e. from around + 40 mV to close to neutrality. Both parameters were about the same as was measured for CPP-coated gold nanorods [56] and ascribed to an interplay between the anionic PEG strands and cationic overcoating. Instead, according to ref 29, MUTAB-coated gold nanorods were a little smaller, on average, and retained more positive polarization in ultrapure water, around + 20 mV. Table 1 recapitulates the hydrodynamic and electrokinetic parameters of the batches compared in this work.

**Table 1:** Summary of the hydrodynamic and electrokinetic parameters of the batches used in this work: polydispersity index (PDI) according to ISO 22412:2008, hydrodynamic diameter (Ø) and electrokinetic potential (ζ)

Parameter	MUTAB	CPP	pLys
PDI	.46 ± .02	.47 ± .04	.54 ± .04
Ø (nm)	65 ± 8	70 ± 7	73 ± 8
ζ (mV)	20 ± 2	6 ± 4	4 ± 3

Figure 1f shows the appearance of murine macrophages that were treated with pLys-coated particles at a concentration of 400 µM Au for a duration of 24 h. All cells display their typical morphology and a constellation of dark organelles that are located inside the cytoplasm in the perinuclear region, which are interpreted as endosomal vesicles containing a high density of gold nanorods. The phagocytic process seems to occur through the extension of pseudopodia.

**Fig. 2:**
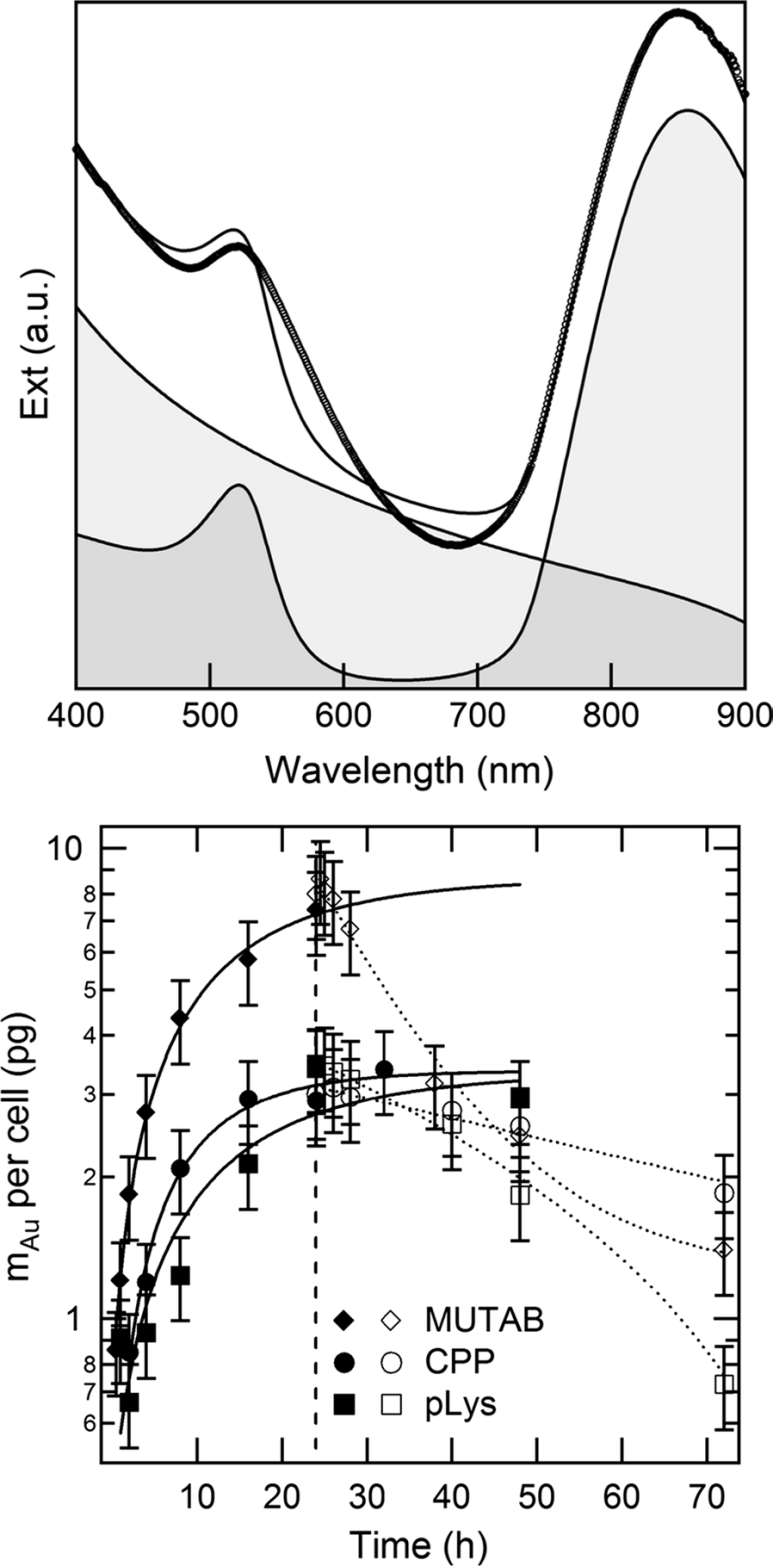
Upper panel: spectrum of optical extinction from a suspension of macrophages exposed to 400 µM Au pLys-coated gold nanorods for 24 h (circlets), together with its numerical fit (line) and corresponding decomposition into an empirical contribution from the macrophages plus an analytical approximation of the plasmonic band of a population of gold nanorods (shadows) [65, 66]. This decomposition was processed to quantify the amount of gold per cell. Lower panel: mass of gold taken up per cell for murine macrophages treated with a dosage of 400 µM Au, during incubation with gold nanorods (full symbols) as well as after their medium was replaced with fresh SFM without particles (empty symbols). The vertical dashed line denotes the timepoint when the replacement took place. Solid and dotted lines are guides to the eye only

Crucial features for the performance of a biological taxi of gold nanorods are the amount of gold taken up per cell during its loading and then retained until arrival at its target site, which may typically take several hours to a few days [68–71]. The upper panel of Fig. 2 displays a spectrum of optical extinction of macrophages treated with pLys-coated particles at a concentration of 400 µM Au for a duration of 24 h and provides an illustration of the method used to quantify their load of gold per cell, by decomposition into the Mie-like contribution from the macrophages, dominating towards the blue/near ultraviolet end, plus the plasmonic fingerprints of the gold nanorods, peaking in the green and red/near infrared windows. A comparison between the optical spectra in Figs. 1, 2 shows that, after being phagocytized, gold nanorods maintained most of their plasmonic features [56, 72]. Some broadening and red-shift of both bands is ascribed to the effect of plasmonic coupling of nearby particles that occurs upon endosomal confinement [53, 73]. The lower panel of Fig. 2 compares the efficiency of internalization and retention of different particles for a representative dosage of 400 µM Au. As for our prior models, MUTAB-coated gold nanorods show faster kinetics of cellular uptake, with a distinct coloration that emerges after as early as a few tens of minutes, with respect to CPP-coated gold nanorods, which take at least 2 h. The accumulation of MUTAB- or CPP-coated particles reaches a plateau around 8 or 3 pg Au per cell, respectively, after about 24 h of co-incubation. However, the advantage of MUTAB- over CPP-coated particles is dissipated after around 24 h of removal of the particles, supposedly because of their difference of exocytic rate. While the origin of this difference will require more investigation, we hypothesize a correlation with the speed and extent of cellular uptake. Moreover, one of the principal functions of macrophages being the elimination of toxic compounds and waste products, the presence of quaternary ammonium compounds may prompt a faster rejection of MUTAB- rather than CPP-coated particles. This interpretation is consistent with the data on cytotoxicity that are reported below.

The kinetics of accumulation and exocytic release of pLys-coated gold nanorods are similar or only slightly worse than the case of CPPs, at least until 24 h of preparation. We conjecture that the kinetics of cellular uptake may increase with the electrokinetic potential in ultrapure water, which is more positive for MUTAB- than CPP- and pLys-coated particles, although the probable formation of a protein corona [74] in a culture medium may preclude any immediate correlation.

We note that the difference from model to model is rather small, especially when macrophages are loaded for 24 h and then assessed after another 24 h of preparation, which is a plausible timeframe for their application, until homing to the TME. The same trend is observed for a lower dosage of gold nanorods (see Additional file 1: Figure S1). When their concentration is reduced from 400 to 100 µM Au, the amount of gold per cell roughly scales by a factor of 3 during preparation and a factor of 2 after 24 h of removal of the particles, when all systems display similar performances.

### Viability of the macrophages

**Fig. 3:**
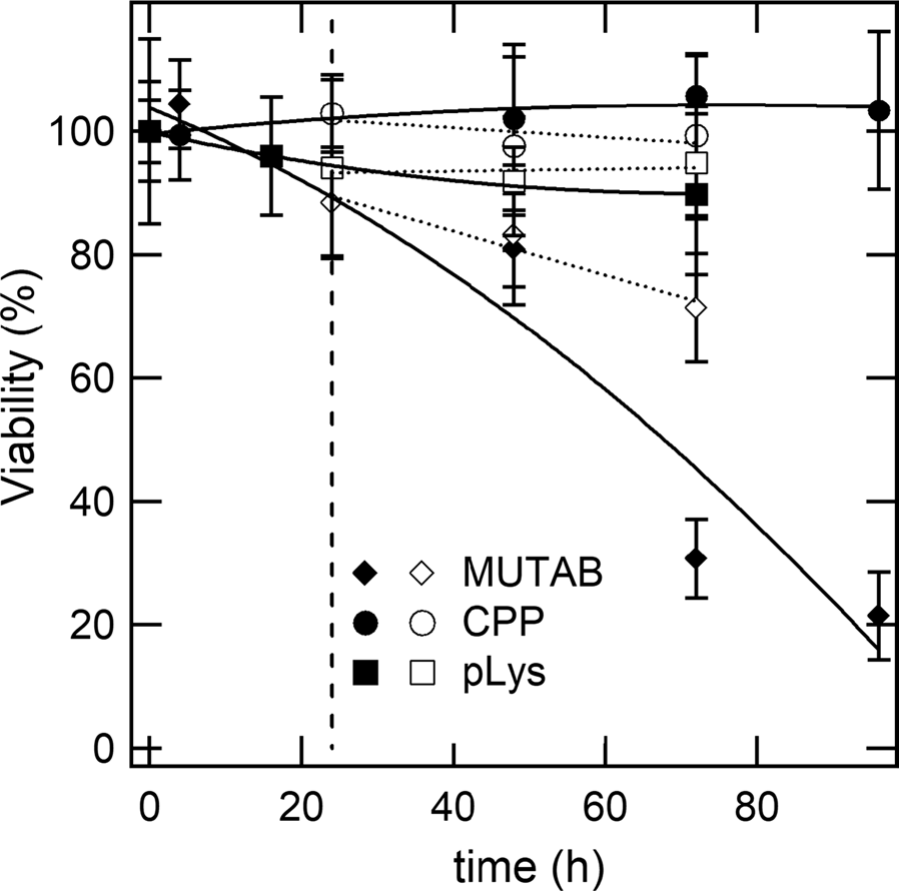
Viability of murine macrophages treated with a dosage of 400 µM Au, in the presence of gold nanorods (full symbols) as well as after their medium was replaced with fresh SFM without particles (empty symbols). The vertical dashed line denotes the timepoint when the replacement took place. Solid and dotted lines are guides to the eye only. Values are expressed as percent of formazan in treated cells vs. untreated controls. Data are reported as mean ± SD of three independent experiments

Another key parameter for the feasibility of a biological taxi of gold nanorods is its viability both during, i.e. the standard cytotoxicity of the particles, as well as after its loading, until arrival at its target site. Figure 3 compares this parameter for different particles at a representative dosage of 400 µM Au.

No significant loss of cell viability was observed in samples treated with CPP- and pLys-coated particles. The fraction of viable cells remains well above 80% even after three or 4 days of co-incubation. Additional file 1 provides complementary data on the proliferation and apoptosis of macrophages treated with pLys-coated gold nanorods for up to 3 days, which corroborate their lack of cytotoxicity. Instead, the cytotoxicity of MUTAB-coated gold nanorods becomes serious after about 2 days and as much as 80% of macrophages are extinct after 4 days. However, relevant vehicles that are loaded for 24 h and then left at rest without particles for up to another 48 h retain around 70% of their pristine viability, which is still hopeful, in view of their application, i.e. until homing to the TME. The viability of macrophages that are treated with CPP- or pLys-coated particles remains rather indistinguishable from that of untreated controls over the entire range of conditions that we have assessed.

That the cytotoxicity of MUTAB-coated particles does not directly relate to their higher uptake is confirmed by a comparison between Fig. 2, Additional file 1: Figures S1, S2, which summarize our full dataset on the viability of macrophages treated under different conditions. Notice, for instance, that the accumulation of MUTAB-coated particles at a dosage of 100 µM Au is slightly lower than that of their CPP-coated counterpart at a dosage of 400 µM Au, but the cytotoxicity of the former is much worse than that of the latter. Instead, we ascribe the cytotoxicity of MUTAB-coated particles to their decoration with quaternary ammonium compounds that are biocidal [75–78] and/or a larger incidence of residual CTAB left after purification [45, 56, 79].

### Chemotaxis and release of cytokines from the macrophages

**Fig. 4:**
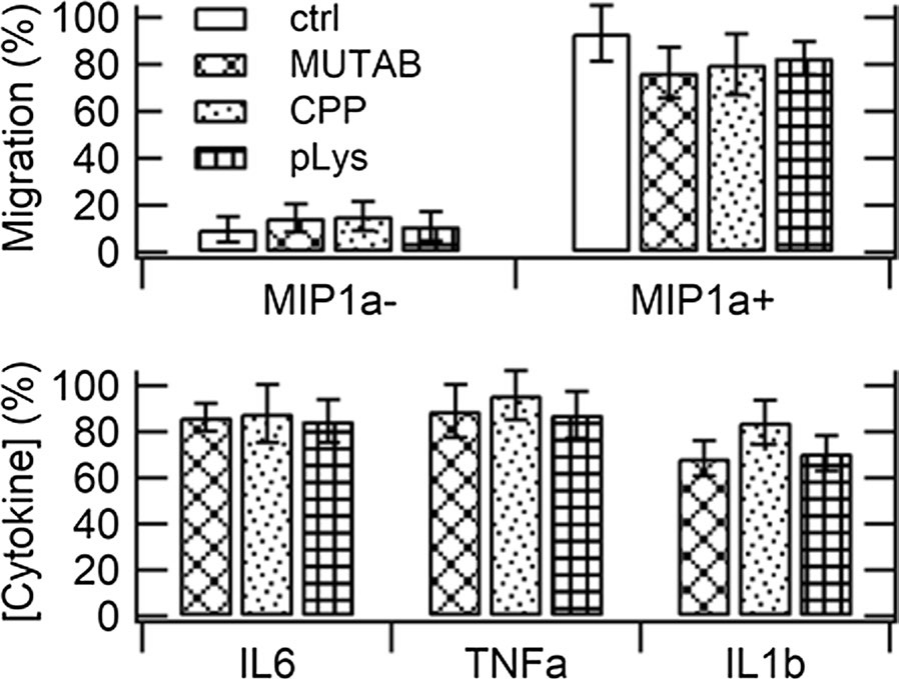
Migration (upper panel) and release of pro-inflammatory cytokines (lower panel) from macrophages loaded with gold nanorods by incubation with a dosage of 400 µM Au for a period of 24 h and then exposed to specific pro-inflammatory stimuli for another period of 18 and 24 h, respectively. The quantification of cytokines in the samples treated with particles is expressed as percent of untreated controls, always in the presence of LPS (no cytokines were found in the absence of LPS). Results are shown as mean ± SD of three independent experiments

In order to gain functional insight into the feasibility of a biological taxi of gold nanorods, we assessed its ability to migrate towards a chemokinic source and to release pro-inflammatory cytokines upon specific stimulation, thus simulating its homing and recruitment of peers in the TME. We have focused on a representative timeframe where the macrophages were fed with the particles for 24 h and then left in the presence of gradients of MIP-1α for 18 h for the observation of their chemotactic profiles or incubated with LPS for 24 h before quantification of their release of cytokines. Results are displayed in Fig. 4.

In the absence of MIP-1α, both relevant controls and macrophages loaded with gold nanorods exhibited little motility. Instead, under a gradient of MIP-1α, a consistent level of cell migration occurred in all samples. Therefore, neither model of particles interfered with the chemotactic profiles of their taxi, which maintained its native potential to home to the TME. In the absence of LPS, the levels of production of cytokines remained null in all samples. Conversely, as occurs in relevant controls, J774a.1 cells loaded with gold nanorods and exposed to LPS underwent activation and released higher levels of IL-6, intermediate levels of TNF-α and lower levels of IL-1β. Therefore, all models of particles exerted little effect on the pro-inflammatory functions of their taxi, which retained its inherent ability to attract peers in the TME. Overall, we found a possible slight correlation between these parameters and cell viability, which is unsurprising. Additional file 1: Figure S3 shows that the same conclusion applies to macrophages that were treated with a lower dosage of gold nanorods and exhibited performances that were even closer to those of relevant controls. Therefore, these particles alone are not able to activate nor inhibit any chemotactic or pro-inflammatory response in macrophages.

### Multiparametric comparison of the particles

The performances of all models of particles are encouraging. Table 2 provides a qualitative summary of our functional survey on the integration of gold nanorods into tumor-tropic macrophages.

**Table 2:** Comparison of different models of cationic coatings for the preparation of a biological taxi of gold nanorods

Parameter	MUTAB	CPP	pLys
Uptake	+++	++	++
Retention	+	+++	++
Viability	+	+++	++
Migration	++	++	+++
Release of cytokines	++	+++	++
Cost-effectiveness	++	+	+++

The symbol + refers to qualitative levels: + = lowest, ++ = intermediate, +++ = highest

Overall, we suggest that MUTAB-coated particles may be more preferable than CPP- or pLys-coated particles for applications that entail a faster delivery, say in the order of a few minutes, and viceversa, because their efficiency of endocytic uptake is fairly better but their kinetics of exocytic release is much faster and their biological profiles are quite worse over longer timescales. Furthermore, the residual cytotoxicity of MUTAB-coated particles may represent a limitation in view of their exploitation. We found that pLys-coated particles are the same or only slightly worse than their CPP-coated predecessors. Both these cases are clearly atoxic, undergo decent cellular uptake and slow excretion over time and do not interfere with cellular functions that are critical for the feasibility of a cell-based delivery system to target the TME. What makes pLys more attractive is its commercial value, which is a fraction of that of CPPs and does not impact much on the overall fabrication of the system.

## Conclusions

We have addressed the modification of gold nanorods as passenger particles for efficient coupling to tumor-tropic cells that may undertake their chemotactic delivery to a tumor site as a biological taxi, for imaging and therapeutic applications. The comparison between MUTAB-, CPP- and pLys-coated gold nanorods reveals favorable features that make these systems a promising platform for translational developments. From a physiological point of view, we have not identified any adverse effect of the use of CPP- and pLys- coated particles on the chemotactic and pro-inflammatory functions of their biological vehicles. On the other hand, MUTAB-coated particles display faster kinetics of cellular uptake, at the expense of greater cytotoxicity and exocytic rates. However, we anticipate that these parameters may improve with incremental refinement of all protocols, including the steps of purification [79], as well as the choice of alternative kinds of tumor-tropic cells, as is under investigation.

The remarkable resemblance of CPP- and pLys-coated gold nanorods suggests that the key requirements for the preparation of passenger particles for efficient coupling to a biological taxi are a handful of metrics that include steric hindrance, cationic profile and a toxicity. This observation represents a terrific simplification with respect to more conventional strategies for the systemic delivery of free particles and may shift the focus of future research onto translational issues, such as regulatory constraints, standardization and costs. Therefore, we are confident that this work will inspire new efforts to bring biological vehicles of functional particles to clinical fruition.

## Additional file

**Additional file 1.** Additional figures.

## References

[1] Kim SW, Khang D (2015). Multiple cues on the physiochemical, mesenchymal, and intracellular trafficking interactions with nanocarriers to maximize tumor target efficiency. Int J Nanomed.

[2] Tan S, Wu T, Zhang D, Zhang Z (2015). Cell or cell membrane-based drug delivery systems. Theranostics.

[3] Pelaz B, Alexiou C, Alvarez-Puebla RA, Alves F, Andrews AM, Ashraf S, Balogh LP, Ballerini L, Bestetti A, Brendel C, Bosi S, Carril M, Chan WC, Chen C, Chen X, Chen X, Cheng Z, Cui D, Du J, Dullin C, Escudero A, Feliu N, Gao M, George M, Gogotsi Y, Grünweller A, Gu Z, Halas NJ, Hampp N, Hartmann RK, Hersam MC, Hunziker P, Jian J, Jiang X, Jungebluth P, Kadhiresan P, Kataoka K, Khademhosseini A, Kopeček J, Kotov NA, Krug HF, Lee DS, Lehr CM, Leong KW, Liang XJ, Ling Lim M, Liz-Marzán LM, Ma X, Macchiarini P, Meng H, Möhwald H, Mulvaney P, Nel AE, Nie S, Nordlander P, Okano T, Oliveira J, Park TH, Penner RM, Prato M, Puntes V, Rotello VM, Samarakoon A, Schaak RE, Shen Y, Sjöqvist S, Skirtach AG, Soliman MG, Stevens MM, Sung HW, Tang BZ, Tietze R, Udugama BN, VanEpps JS, Weil T, Weiss PS, Willner I, Wu Y, Yang L, Yue Z, Zhang Q, Zhang Q, Zhang XE, Zhao Y, Zhou X, Parak WJ (2017). Diverse applications of nanomedicine. ACS Nano.

[4] Greish K (2007). Enhanced permeability and retention of macromolecular drugs in solid tumors: a royal gate for targeted anticancer nanomedicines. J Drug Target.

[5] Perrault SD, Walkey C, Jennings T, Fischer HC, Chan WC (2009). Mediating tumor targeting efficiency of nanoparticles through design. Nano Lett.

[6] Danhier F, Feron O, Préat V (2010). To exploit the tumor microenvironment: Passive and active tumor targeting of nanocarriers for anti-cancer drug delivery. J Control Release.

[7] Prabhakar U, Maeda H, Jain RK, Sevick-Muraca EM, Zamboni W, Farokhzad OC, Barry ST, Gabizon A, Grodzinski P, Blakey DC (2013). Challenges and key considerations of the enhanced permeability and retention (EPR) effect for nanomedicine drug delivery in oncology. Cancer Res.

[8] El-Sayed IH, Huang X, El-Sayed MA (2005). Surface plasmon resonance scattering and absorption of anti-EGFR antibody conjugated gold nanoparticles in cancer diagnostics: applications in oral cancer. Nano Lett.

[9] Byrne JD, Betancourt T, Brannon-Peppas L (2008). Active targeting schemes for nanoparticle systems in cancer therapeutics. Adv Drug Deliv Rev.

[10] Huang X, Peng X, Wang Y, Wang Y, Shin DM, El-Sayed MA, Nie S (2010). A reexamination of active and passive tumor targeting by using rod-shaped gold nanocrystals and covalently conjugated peptide ligands. ACS Nano.

[11] Dreaden EC, Austin LA, Mackey MA, El-Sayed MA (2012). Size matters: gold nanoparticles in targeted cancer drug delivery. Ther Deliv.

[12] Siveen KS, Kuttan G (2009). Role of macrophages in tumour progression. Immunol Lett.

[13] Barcellos-de-Souza P, Gori V, Bambi F, Chiarugi P (2013). Tumor microenvironment: bone marrow-mesenchymal stem cells as key players. Biochim Biophys Acta.

[14] Chiarugi P, Paoli P, Cirri P (2014). Tumor microenvironment and metabolism in prostate cancer. Semin Oncol.

[15] Comito G, Giannoni E, Segura CP, Barcellos-de-Souza P, Raspollini MR, Baroni G, Lanciotti M, Serni S, Chiarugi P (2014). Cancer-associated fibroblasts and M2-polarized macrophages synergize during prostate carcinoma progression. Oncogene.

[16] Barcellos-de-Souza P, Comito G, Pons-Segura C, Taddei ML, Gori V, Becherucci V, Bambi F, Margheri F, Laurenzana A, Del Rosso M, Chiarugi P (2016). Mesenchymal stem cells are recruited and activated into carcinoma-associated fibroblasts by prostate cancer microenvironment-derived TGF-β1. Stem Cells.

[17] Choi MR, Stanton-Maxey KJ, Stanley JK, Levin CS, Bardhan R, Akin D, Badve S, Sturgis J, Robinson JP, Bashir R, Halas NJ, Clare SE (2007). A cellular Trojan horse for delivery of therapeutic nanoparticles into tumors. Nano Lett.

[18] Holden CA, Yuan Q, Yeudall WA, Lebman DA, Yang H (2009). Surface engineering of macrophages with nanoparticles to generate a cell-nanoparticle hybrid vehicle for hypoxia-targeted drug delivery. Int J Nanomed.

[19] Beard P (2011). Biomedical photoacoustic imaging. Interface Focus.

[20] Li WW, Chen XY (2015). Gold nanoparticles for photoacoustic imaging. Nanomedicine.

[21] Weber J, Beard PC, Bohndiek SE (2016). Contrast agents for molecular photoacoustic imaging. Nat Methods.

[22] Jiang YY, Pu KY (2017). Advanced photoacoustic imaging applications of near-infrared absorbing organic nanoparticles. Small.

[23] Svaasand LO, Gomer CJ, Morinelli E (1990). On the physical rationale of laser induced hyperthermia. Lasers Med Sci.

[24] Huang XH, Jain PK, El-Sayed IH, El-Sayed MA (2008). Plasmonic photothermal therapy (PPTT) using gold nanoparticles. Lasers Med Sci.

[25] Chatterjee DK, Diagaradjane P, Krishnan S (2011). Nanoparticle-mediated hyperthermia in cancer therapy. Ther Deliv..

[26] Zou LL, Wang H, He B, Zeng LJ, Tan T, Cao HQ, He XY, Zhang ZW, Guo SR, Li YP (2016). Current approaches of photothermal therapy in treating cancer metastasis with nanotherapeutics. Theranostics.

[27] Baek SK, Makkouk AR, Krasieva T, Sun CH, Madsen SJ, Hirschberg H (2011). Photothermal treatment of glioma; an in vitro study of macrophage-mediated delivery of gold nanoshells. J Neurooncol.

[28] Cavigli L, Tatini F, Borri C, Ratto F, Centi S, Cini A, Lelli B, Matteini P, Pini R (2016). Preparation and photoacoustic analysis of cellular vehicles containing gold nanorods. J Vis Exp..

[29] Ratto F, Centi S, Avigo C, Borri C, Tatini F, Cavigli L, Kusmic C, Lelli B, Lai S, Benagiano M, D’Elios MM, Colagrande S, Faita F, Menichetti L, Pini R (2016). A robust design for cellular vehicles of gold nanorods for multimodal imaging. Adv Funct Mater.

[30] Kennedy LC, Bear AS, Young JK, Lewinski NA, Kim J, Foster AE, Drezek RA (2011). T cells enhance gold nanoparticle delivery to tumors in vivo. Nanoscale Res Lett.

[31] Baldi G, Ravagli C, Comes Franchini M, D’Elios MM, Benagiano M, Bitossi M (Colorobbia Italia S.p.A.). Magnetic nanoparticles functionalized with cathecol, production and use thereof. *WO2015104664 A1*, 2015.

[32] Kang S, Bhang SH, Hwang S, Yoon JK, Song J, Jang HK, Kim S, Kim BS (2015). Mesenchymal stem cells aggregate and deliver gold nanoparticles to tumors for photothermal therapy. ACS Nano.

[33] Encabo-Berzosa MM, Gimeno M, Lujan L, Sancho-Albero M, Gomez L, Sebastian V, Quintanilla M, Arruebo M, Santamaria J, Martin-Duque M (2016). Selective delivery of photothermal nanoparticles to tumors using mesenchymal stem cells as Trojan horses. RSC Adv..

[34] Schnarr K, Mooney R, Weng Y, Zhao D, Garcia E, Armstrong B, Annala AJ, Kim SU, Aboody KS, Berlin JM (2013). Gold nanoparticle-loaded neural stem cells for photothermal ablation of cancer. Adv Healthc Mater.

[35] Mooney R, Roma L, Zhao DH, Van Haute D, Garcia E, Kim SU, Annala AJ, Aboody KS, Berlin JM (2014). Neural stem cell-mediated intratumoral delivery of gold nanorods improves photothermal therapy. ACS Nano.

[36] Margheri G, Zoppi A, Olmi R, Trigari S, Traversi R, Severi M, Bani D, Bianchini F, Torre E, Margheri F, Chillà A, Biagioni A, Calorini L, Laurenzana A, Fibbi G, Del Rosso M (2016). Tumor-tropic endothelial colony forming cells (ECFCs) loaded with near-infrared sensitive Au nanoparticles: a “cellular stove” approach to the photoablation of melanoma. Oncotarget.

[37] Bauer LA, Birenbauma NS, Meyer GJ (2004). Biological applications of high aspect ratio nanoparticles. J Mater Chem.

[38] Dreaden EC, Alkilany AM, Huang X, Murphy CJ, El-Sayed MA (2012). The golden age: gold nanoparticles for biomedicine. Chem Soc Rev.

[39] Pérez-Juste J, Pastoriza-Santos I, Liz-Marzán LM, Mulvaney P (2005). Gold nanorods: synthesis, characterization and applications. Coord Chem Rev.

[40] Ratto F, Matteini P, Rossi F, Pini R (2010). Size and shape control in the overgrowth of gold nanorods. J Nanopart Res.

[41] Vigderman L, Khanal BP, Zubarev ER (2012). Functional gold nanorods: synthesis, self-assembly, and sensing applications. Adv Mater.

[42] Chen HJ, Shao L, Li Q, Wang JF (2013). Gold nanorods and their plasmonic properties. Chem Soc Rev.

[43] DeBrosse MC, Comfort KK, Untener EA, Comfort DA, Hussain SM (2013). High aspect ratio gold nanorods displayed augmented cellular internalization and surface chemistry mediated cytotoxicity. Mater Sci Eng C.

[44] Niidome T, Yamagata M, Okamoto Y, Akiyama Y, Takahashi H, Kawano T, Katayama Y, Niidome Y (2006). PEG-modified gold nanorods with a stealth character for in vivo applications. J Control Release.

[45] Tatini F, Landini I, Scaletti F, Massai L, Centi S, Ratto F, Nobili S, Romano G, Fusi F, Messori L, Mini E, Pini R (2014). Size dependent biological profiles of PEGylated gold nanorods. J Mater Chem B.

[46] Vigderman L, Manna P, Zubarev ER (2012). Quantitative replacement of cetyl trimethylammonium bromide by cationic thiol ligands on the surface of gold nanorods and their extremely large uptake by cancer cells. Angew Chem.

[47] Troiano JM, Olenick LL, Kuech TR, Melby ES, Hu D, Lohse SE, Mensch A, Dogangun M, Vartanian AM, Torelli MD, Ehimiaghe E, Walter SR, Fu L, Anderton CR, Zhu Z, Wang H, Orr G, Murphy CJ, Hamers RJ, Pedersen JA, Geiger FM (2015). Direct probes of 4 nm diameter gold nanoparticles interacting with supported lipid bilayers. J Phys Chem C.

[48] Melby ES, Mensch AC, Lohse SE, Hu D, Orr G, Murphy CJ, Hamers RJ, Pedersen JA (2016). Formation of supported lipid bilayers containing phase-segregated domains and their interaction with gold nanoparticles. Environ Sci Nano..

[49] Troiano JM, Kuech TR, Vartanian AM, Torelli MD, Sen A, Jacob LM, Hamers RJ, Murphy CJ, Pedersen JA, Geiger FM (2016). On electronic and charge interference in second harmonic generation responses from gold metal nanoparticles at supported lipid bilayers. J Phys Chem C.

[50] Dobrovolskaia MA, Patri AK, Zheng J, Clogston JD, Ayub N, Aggarwal P, Neun BW, Hall JB, McNeil SE (2009). Interaction of colloidal gold nanoparticles with human blood: effects on particle size and analysis of plasma protein binding profiles. Nanomedicine.

[51] Wang P, Wang X, Wang L, Hou X, Liu W, Chen C (2015). Interaction of gold nanoparticles with proteins and cells. Sci Technol Adv Mater.

[52] Arro L, Salenstedt CR (1973). Evaluation of toxicity of some quaternary ammonium compounds. J Biol Stand..

[53] Tezel U (2009). Fate and effect of quaternary ammonium compounds in biological systems.

[54] Inácio AS, Costa GN, Domingues NS, Santos MS, Moreno AJM, Vaz WLC, Vieira OV (2013). Mitochondrial dysfunction is the focus of quaternary ammonium surfactant toxicity to mammalian epithelial cells. Antimicrob Agents Chemother.

[55] Lavorgna M, Russo C, D’Abrosca B, Parrella A, Isidori M (2016). Toxicity and genotoxicity of the quaternary ammonium compound benzalkonium chloride (BAC) using *Daphnia magna* and *Ceriodaphnia dubia* as model systems. Environ Pollut.

[56] Lai S, Centi S, Borri C, Ratto F, Cavigli L, Micheletti F, Kemper B, Ketelhut S, Kozyreva T, Gonnelli L, Rossi F, Colagrande S, Pini R (2017). A multifunctional organosilica cross-linker for the bio-conjugation of gold nanorods. Colloids Surf B.

[57] Sebbage V (2009). Cell-penetrating peptides and their therapeutic applications. Biosci Horizons.

[58] Copolovici DM, Langel K, Eriste E, Langel U (2014). Cell-penetrating peptides: design, synthesis, and applications. ACS Nano.

[59] Dinca A, Chien WM, Chin MT (2016). Intracellular delivery of proteins with cell-penetrating peptides for therapeutic uses in human disease. Int J Mol Sci.

[60] Guidotti G, Brambila L, Rossi D (2017). Cell-penetrating peptides: from basic research to clinics. Trends Pharmacol Sci.

[61] Liu M, Zhang X, Yang B, Liu L, Deng F, Zhang X, Wei Y (2014). Polylysine crosslinked AIE dye based fluorescent organic nanoparticles for biological imaging applications. Macromol Biosci.

[62] Chen X, Kube DM, Cooper MJ, Davis PB (2008). Cell surface nucleolin serves as receptor for DNA nanoparticles composed of pegylated polylysine and DNA. Mol Ther.

[63] Nikoobakht B, El-Sayed MA (2003). Preparation and growth mechanism of gold nanorods (NRs) using seed-mediated growth method. Chem Mater.

[64] Etchegoin PG, Le Ru EC, Meyer M (2006). An analytic model for the optical properties of gold. J Chem Phys..

[65] Ratto F, Matteini P, Cini A, Centi S, Rossi F, Fusi F, Pini R (2011). CW laser-induced photothermal conversion and shape transformation of gold nanodogbones in hydrated chitosan films. J Nanopart. Res..

[66] Ratto F, Witort E, Tatini F, Centi S, Lazzeri L, Carta F, Lulli M, Vullo D, Fusi F, Supuran CT, Scozzafava A, Capaccioli S, Pini R (2015). Plasmonic particles that hit hypoxic cells. Adv Funct Mater.

[67] Centi S, Tatini F, Ratto F, Gnerucci A, Mercatelli R, Romano G, Landini I, Nobili S, Ravalli A, Marrazza G, Mini E, Fusi F, Pini R (2014). In vitro assessment of antibody-conjugated gold nanorods for systemic injections. J Nanobiotechnol.

[68] Chithrani BD, Chan WC (2007). Elucidating the mechanism of cellular uptake and removal of protein-coated gold nanoparticles of different sizes and shapes. Nano Lett.

[69] Nativo P, Prior IA, Brust M (2008). Uptake and intracellular fate of surface-modified gold nanoparticles. ACS Nano.

[70] Alkilany AM, Murphy CJ (2010). Toxicity and cellular uptake of gold nanoparticles: what we have learned so far?. J Nanopart Res.

[71] Rothen-Rutishauser B, Kuhn D, Ali Z, Gasser M, Amin F, Parak W, Vanhecke D, Petri-Fink A, Gehr P, Brandenberger C (2013). Quantification of gold nanoparticle cell uptake under controlled biological conditions and adequate resolution. Nanomedicine.

[72] Cavigli L, Cini A, Centi S, Borri C, Lai S, Ratto F, de Angelis M, Pini R (2017). Photostability of gold nanorods upon endosomal confinement in cultured cells. J Phys Chem C.

[73] Mazzoni M, Ratto F, Fortunato C, Centi S, Tatini F, Pini R (2014). Partial decoupling in aggregates of silanized gold nanorods. J Phys Chem C.

[74] Walkey CD, Chan WC (2012). Understanding and controlling the interaction of nanomaterials with proteins in a physiological environment. Chem Soc Rev.

[75] Moriarty TF, Zaat SAJ, Busscher HJ (2012). Biomaterials associated infection: immunological aspects and antimicrobial strategies.

[76] Melin VE, Potineni H, Hunt P, Griswold J, Siems B, Werre SR, Hrubec TC (2014). Exposure to common quaternary ammonium disinfectants decreases fertility in mice. Reprod Toxicol..

[77] Stupar M, Grbić ML, Džamić A, Unković N, Ristić M, Jelikić A, Vukojević J (2014). Antifungal activity of selected essential oils and biocide benzalkonium chloride against the fungi isolated from cultural heritage objects. S Afr J Bot.

[78] Gerba CP (2015). Quaternary ammonium biocides: efficacy in application. Appl Environ Microbiol.

[79] Kinnear C, Dietsch H, Clift MJD, Endes C, Rothen-Rutishauser B, Petri-Fink A (2013). Gold nanorods: controlling their surface chemistry and complete detoxification by a two-step place exchange. Angew Chem.

