# Supplementary material for: Polylysine as a functional biopolymer to couple gold nanorods to tumor-tropic cells
Source: J Nanobiotechnology. 2018 May 31;16:50. doi: 10.1186/s12951-018-0377-7 (PMC5984317; doi:10.1186/s12951-018-0377-7)
Supplement: Supplementary file 1 — Additional file 1. Additional figures. [file 12951_2018_377_MOESM1_ESM.docx]

Additional File 1


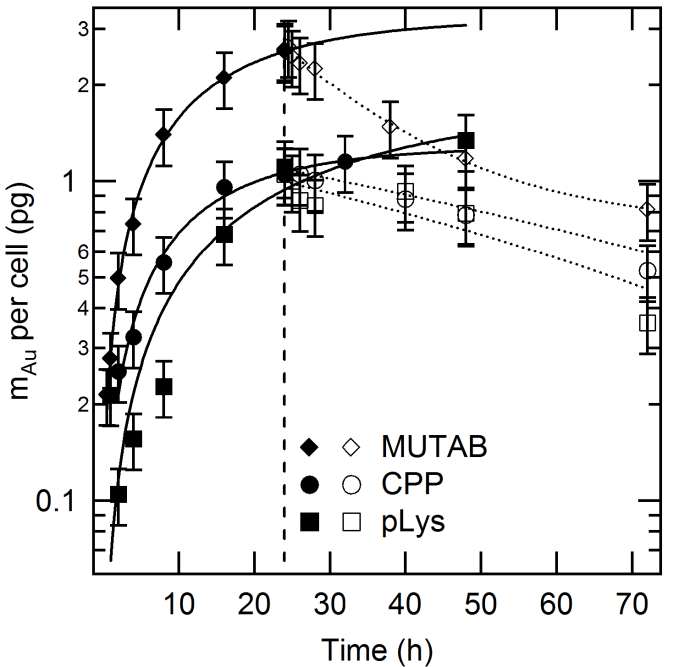


Additional file 1: Figure S1: mass of gold taken up per cell for murine macrophages treated with a dosage of 100 µM Au, during incubation with gold nanorods (full symbols) as well as after their medium was replaced with fresh SFM without particles (empty symbols). The vertical dashed line denotes the timepoint when the replacement took place. Solid and dotted lines are guides to the eye only.


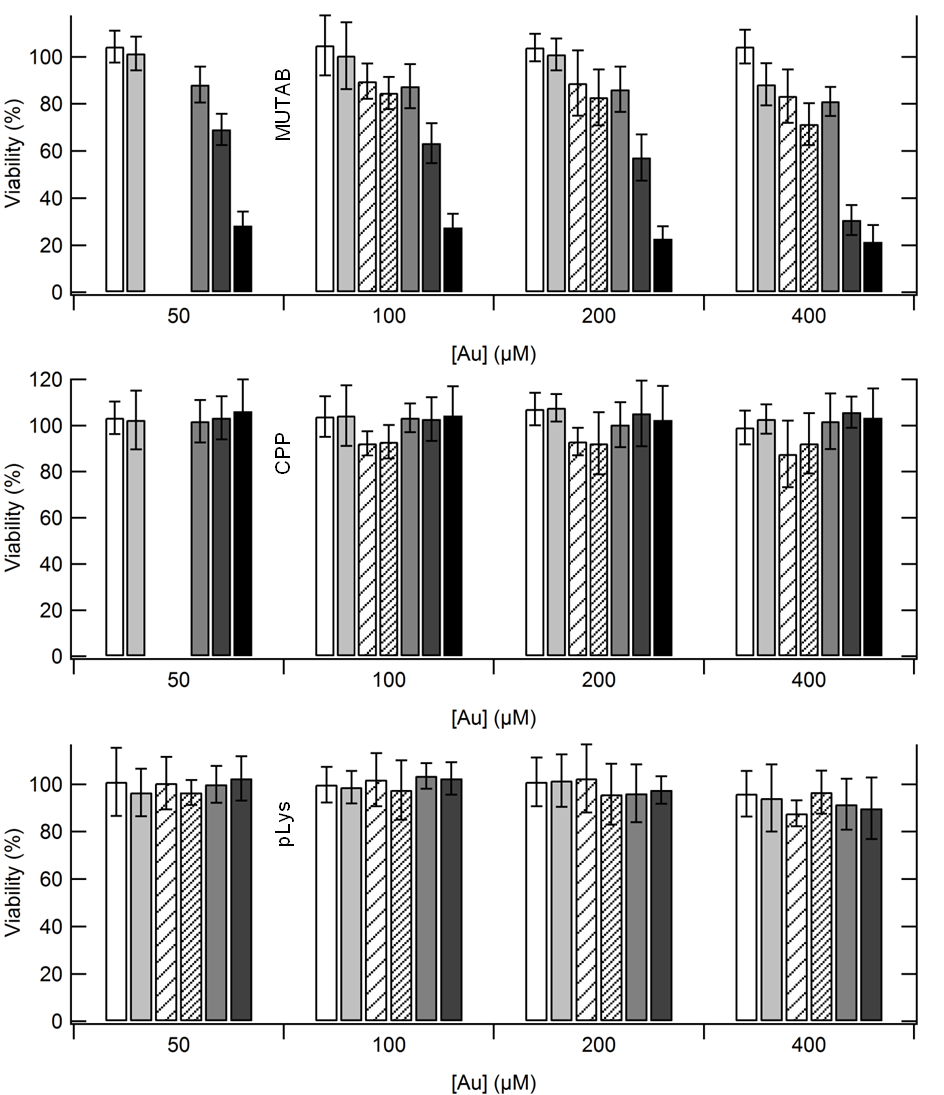


Additional file 1: Figure S2: viability of murine macrophages incubated with gold nanorods (full bars) for different timepoints as well as 24 (less shadowed bars) or 48 hrs (more shadowed bars) past the removal of the particles dosed at the named concentrations for 24 hrs. As for the full bars, the grey scale goes, from white to dark, from 4 to 24, 48, 72 and 96 hrs for MUTAB- and CPP-coated particles and from 16 to 24, 48 and 72 hrs for pLys-coated particles. Values are expressed as percent of WST-8 reduction in treated cells vs. untreated controls. Data are reported as mean ± SD of three independent experiments.


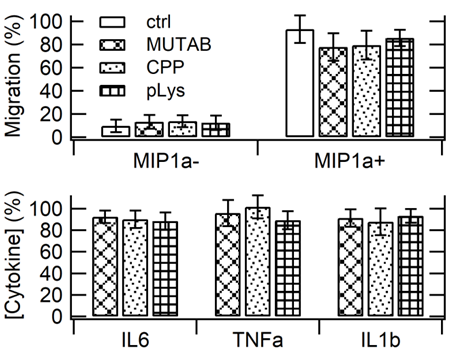


Additional file 1: Figure S3: migration (upper panel) and release of pro-inflammatory cytokines (lower panel) from biological vehicles of gold nanorods prepared upon incubation with a dosage of 100 µM Au for a period of 24 hrs and then exposed to specific pro-inflammatory stimuli for another period of 18 and 24 hrs, respectively. The quantification of cytokines in the samples treated with particles is expressed as percent of untreated controls, always in the presence of LPS (no cytokines were found in the absence of LPS). Results are shown as mean ± SD of three independent experiments.

*Proliferation assay by Sulforhodamine B*

In order to corroborate the biocompatibility of pLys-coated gold nanorods, the cellular proliferation was assessed by a Sulforhodamine B (SRB) assay, which provides an inexpensive and accurate quantification of the number of cells per well [80, 81]. This test probes the total biomass by staining cellular proteins. SRB is a bright-pink aminoxanthene dye that forms electrostatic complexes with alkaline amino acid residues under slightly acidic conditions and dissociates under alkaline conditions. Since the number of cells correlates with the amount of internalized dye, this test provides insight into the cytotoxicity of the particles.

8 × 10^3^ cells were cultured in 96-well plates and treated with 50, 100, 200 and 400 µM Au pLys-coated gold nanorods for 24, 48 and 72 hrs in triplicate. At each timepoint, particles were removed and cells were carefully washed and fixed with 50% (w/v) trichloroacetic acid in ultrapure water for one hr at 4°C. Then, cells were rinsed several times with ultrapure water, stained with 0.4% SRB solution in 1% acetic acid for 30 min and finally washed with 1% acetic acid, in order to remove the free dye. The incorporated dye was finally solubilized with 10 mM Tris Base solution for 10 min under gentle stirring and its optical absorbance was quantified by a LT-4000 microplate reader from Labtech at 570 nm with reference at 630 nm. Results were expressed as percent of optical intensity of treated samples versus controls.

As shown in the Figure S4, data correlate with the metabolic activity observed by a WST-8 assay and reported in Fig S2. No statistically significant anti-proliferative effect was detected at any timepoint tested until three days. On the other hand, not even an increase in cellular proliferation was found with respect to controls. Therefore, pLys-coated gold nanorods do not inhibit nor stimulate the replication of murine macrophages.

**
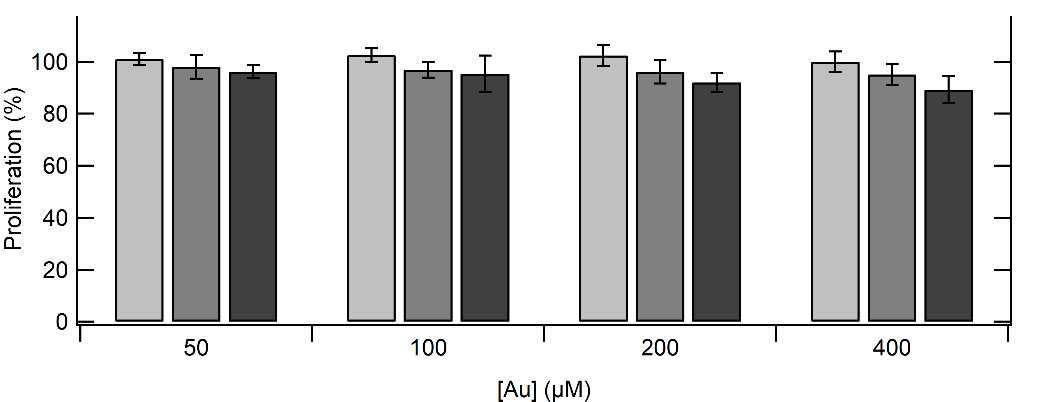
**

Additional file 1: Figure S4: Proliferation of murine macrophages exposed to pLys-coated gold nanorods for 24, 48 and 72 hrs, from brighter to darker. Data are shown as percent of the optical density of treated cells with respect to controls. Results are reported as mean ± SD of three independent experiments.

*Apoptosis detection with DAPI*

Apoptosis is an ATP-dependent process that conveys morphological and biochemical alterations [82, 83]. In particular, apoptotic cells exhibit well-defined morphological features that include maintenance of the integrity, contraction and blebbing of their plasmatic membranes, decrease of their volumes (i.e. cell shrinkage), condensation of chromatin, fragmentation of their nuclei and, in late stages, formation of apoptotic bodies. The main biochemical markers consist of the externalization of phosphatidylserine, the translocation of cytochrome C and pro-apoptotic proteins (e.g. Bax and Bak) and the activation of caspases.

In order to investigate short- and medium-term effects of an exposure to pLys-coated gold nanorods on genetic damage that may induce cellular apoptosis, samples were stained with DAPI (4',6-diamidino-2-phenylindole) and compared to relevant controls. DAPI binds nucleic acids and preferentially double-stranded DNA in AT-rich regions. Based on the intensity of fluorescence and nuclear morphology, it is possible to distinguish between healthy and apoptotic cells [84, 85].

Briefly, 5 × 10^4^ J774a.1 cells were plated in 35-mm Petri dishes featuring a specific polymer coverslip bottom suitable for high-resolution microscopy (µ-Dish 35 mm, high, ibiTreat, ibidi®, Germany) and incubated with 100 and 400 µM Au pLys-coated gold nanorods for 24, 48 and 72 hrs in serum-free medium. After removal of the particles, cells were washed with PBS, fixed and permeabilized with a solution of acetone:methanol (1:1) for 20 min at -20°C. Nuclei were then stained with DAPI for 5 min in the dark. Finally, each sample was excited by a 405 nm diode laser and its blue fluorescence was observed with an SP8 confocal laser scanning microscope from Leica Microsystems (Mannheim, Germany) at 461 nm and 63× magnification.

Controls in Figure S5 display the typical conformation of the nuclei of normal cells [86], which are round and exhibit clear margins and an intense and uniform staining. In contrast, apoptotic cells usually display abnormal nuclear margins, condensed chromosomes and nuclear fragmentation [87]. The appearance of macrophages treated with pLys-coated gold nanorods is similar to controls at all timepoints. Neither DNA fragmentation nor chromatin condensation were noticed in any sample. Therefore, exposure of murine macrophages to pLys-coated gold nanorods does not trigger their programmed death until three days.


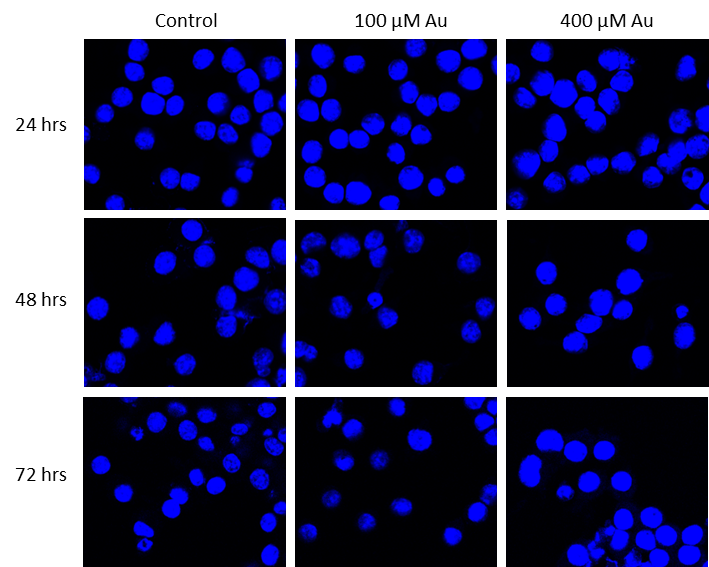


Additional file 1: Figure S5: Representative images of the nuclear morphology of controls and macrophages incubated with 100 and 400 µM Au pLys-coated gold nanorods for 24, 48 and 72 hrs from a confocal microscope at 63× magnification. Nuclei stained with DAPI display an intense blue fluorescence.

In conclusion, we have assessed cell viability, proliferation and apoptosis and we have not found any evidence of a significant cytotoxicity of pLys-coated gold nanorods until a dose of 400 µM Au for up to three days.

Additional references

80. Skehan P, Storeng R, Scudiero D, Monks A, McMahon J, Vistica D, Warren JT, Bokesch H, Kenney S and Boyd MR. New colorimetric cytotoxicity assay for anticancer-drug screening. *J Natl Cancer Inst.* 1990; 82(13): 1107-1112.

81. Keepers YP, Pizao PE, Peters GJ, van Ark-Otte J, Winograd B and Pinedo HM. Comparison of the sulforhodamine B protein and tetrazolium (MTT) assays for in vitro chemosensitivity testing. *Eur J Cancer.* 1991; 27(7): 897-900.

82. Häcker G. The morphology of apoptosis. *Cell Tissue Res.* 2000; 301(1): 5-17. doi: 10.1007/s004410000193.

83. Elmore S. Apoptosis: A review of programmed cell death. *Toxicol Pathol.* 2007; 35(4): 495-516. doi: 10.1080/01926230701320337.

84. Cummings BS, Wills LP and Schnellmann RG. Measurement of cell death in mammalian cells. *Curr Protoc Pharmacol.* 2004; 12: 12.8. doi: 10.1002/0471141755.ph1208s25.

85. Eidet JR, Pasovic L, Maria R, Jackson CJ and Utheim TP. Objective assessment of changes in nuclear morphology and cell distribution following induction of apoptosis. *Diagn Pathol*. 2014; 9: 92. doi: 10.1186/1746-1596-9-92.

86. Webster M, Witkin KL and Cohen-Fix O. Sizing up the nucleus: nuclear shape, size and nuclear-envelope assembly. *J Cell Sci.* 2009; 122(10): 1477-1486. doi: 10.1242/jcs.037333.

87. Rogalińska M. Alterations in cell nuclei during apoptosis. *Cell Mol Biol Lett.* 2002; 7(4): 995-1018.
